# Lower limb biomechanics in individuals with chronic ankle instability during gait: a case-control study

**DOI:** 10.1186/s13047-021-00476-6

**Published:** 2021-05-03

**Authors:** Gabriel Moisan, Camille Mainville, Martin Descarreaux, Vincent Cantin

**Affiliations:** 1 Department of Human Kinetics, Université du Québec à Trois-Rivières, Trois-Rivières, Canada; 2 Groupe de recherche sur les affections neuro-musculo-squelettiques (GRAN), Université du Québec à Trois-Rivières, Trois-Rivières, Canada

**Keywords:** Chronic ankle instability, Gait, Kinematics, Kinetics, Electromyography, Biomechanics

## Abstract

**Background:**

Individuals with chronic ankle instability (CAI) exhibit many biomechanical changes to lower limbs during walking. However, only a few studies have investigated the differences in lower limb biomechanics of individuals with CAI compared to healthy controls using a comprehensive approach including kinematic, kinetic and electromyography (EMG) measures. Consequently, the theoretical framework explaining the biomechanical adaptations in individuals with CAI is mostly based on the results of studies including heterogenous methods and participants’ specificities (e.g., level of disability). More studies using a comprehensive approach are needed to better understand the biomechanical adaptations associated with CAI. The objective of this case-control study was to identify the kinematic, kinetic and EMG differences between individuals with CAI and healthy controls during walking.

**Methods:**

Twenty-eight individuals with CAI and 26 healthy controls were recruited to walk at a self-selected speed during which lower limb kinematics, kinetics and EMG were analysed. Ankle and knee angles and moments as well as gluteus medius, vastus lateralis, gastrocnemius lateralis, peroneus longus and tibialis anterior muscles activity were compared between the CAI and control groups using one-dimensional statistical parametric mapping.

**Results:**

The CAI group exhibited greater ankle inversion angles from 14 to 48% of the stance phase (%SP) (*p* = 0.008), ankle eversion moments from 40 to 78%SP (*p* < 0.001), knee abduction moments from 3 to 6%SP and peroneus longus muscle activity from 0 to 15%SP (*p* = 0.003) and 60 to 76%SP (*p* = 0.003) compared to the control group. No significant between-group differences in ankle sagittal and transverse angles and moments, knee angles, knee sagittal and transverse moments as well as gluteus medius, vastus lateralis, gastrocnemius lateralis and tibialis anterior muscles activity were found.

**Conclusions:**

During the first half of the stance phase, individuals with CAI could be at more risk of sustaining recurrent LAS mostly due to greater ankle inversion angles. However, the greater ankle eversion moments and peroneus longus muscle activity during the second half of the stance phase were an efficient mechanism to correct this maladaptive gait pattern and allowed to attenuate the faulty ankle movements during the pre-swing phase.

**Supplementary Information:**

The online version contains supplementary material available at 10.1186/s13047-021-00476-6.

## Background

Lateral ankle sprains (LAS) are very common in the active population [1] with nearly two million annual reported cases in the United States of America [2]. However, the true incidence of LAS is a lot higher considering that 55 to 64% of individuals sustaining a LAS will not seek professional health care [3, 4]. Seventy percent of individuals will sustain at least one recurrent sprain after the index LAS [5] and 40% will develop chronic ankle instability (CAI) [6]. CAI is characterised by a propensity for recurrent LAS at least 12 months after the index LAS, frequent episodes of ankle giving way, persistent symptoms such as pain, swelling, limited motion, weakness and diminished self-reported function [7]. CAI leads to altered balance control, ankle neuromuscular function, and lower limb biomechanics during locomotion [8–10]. These deficits place individuals with CAI at greater risk of sustaining recurrent sprains [11], developing post-traumatic ankle osteoarthritis [12] and consequently decreasing their health-related quality of life [13].

Since several decades, clinical gait analysis provides objective useful information about the mechanical and neuromuscular deficits in individuals with CAI [8]. During walking, individuals with CAI exhibit greater ankle inversion angles [14–16] which place more load under the lateral part of the foot [17, 18]. These biomechanical deficits could predispose their ankle to give way and sustain a recurrent sprain [8]. To attenuate this cascade of biomechanical events, a greater activity of the peroneus longus muscle, the main evertor of the ankle complex, is observed in these individuals [14, 17, 18] and could represent a protective mechanism [14]. Changes in other lower limb muscles’ activity in individuals with CAI are inconsistent in previous studies [8]. It could be attributed to the spectrum of disability associated with CAI [7] and by the great disparity in reported EMG analyses [8]. There is a need to standardise the processing and normalisation methods in studies investigating EMG of the lower limbs in individuals with CAI to increase the external validity of the published results.

Previous studies reported inconsistent knee biomechanics deficits in individuals with CAI [14–16, 19, 20]. A few studies found no differences between the knee biomechanics of individuals with CAI compared to healthy controls [14, 16, 19]. Also, two recent studies reported conflicting results when comparing knee moments between these two groups [15, 20]. Moisan et al. [20] reported greater and Son et al. [15] reported smaller knee abduction moments in individuals with CAI compared to controls during walking. Further studies are needed to better understand the proximal adaptations to the lower limb biomechanics during walking. Also, only a few studies investigated the biomechanics of the lower limbs of individuals with CAI compared to healthy controls [15, 20] using a comprehensive approach including kinematic, kinetic and EMG measures. Consequently, the theoretical framework explaining the biomechanical adaptations in individuals with CAI is mostly based on the results of studies including heterogeneous methods and participants’ specificities (e.g., level of disability). More studies using a comprehensive approach are needed to identify the biomechanical adaptations associated with CAI for the same population and investigate how kinematic, kinetic and EMG adaptations interact.

The objective of this case-control study was to identify the kinematic, kinetic and EMG differences between individuals with CAI and controls during overground walking. It was hypothesised that individuals with CAI will exhibit greater ankle inversion angles and peroneus longus muscle activity as well as smaller ankle inversion moments compared to controls.

## Methods

### Participants

Fifty-four participants (CAI = 28, controls = 26) were recruited among the staff and students of the Université du Québec à Trois-Rivières (UQTR), Canada, by means of referral from the UQTR outpatient podiatry clinic and via social media advertisements. Sample size was determined, using the preliminary results of this study, with G-Power software (Version 3.1, Kiel, Germany). It was determined that, for ankle frontal angles and moments, at least 50 participants were necessary to obtain a Cohen’s d effect size > 0.50 using alpha< 0.05 and beta> 0.80. To ensure adequate power owing technical difficulties during data acquisition or analyses, 54 participants were included in this study. The used inclusion criteria for the CAI group were based on the recommendations of the International Ankle Consortium [21] except for the confirmation of self-reported ankle instability with a validated questionnaire, as no French versions were available at the time of data collection. Participants with CAI had a history of one or more LAS, ankle giving way and/or recurrent sprains and/or feeling of ankle instability. They had to score less than 90 and 80% at the Foot and Ankle Ability Measures-Activity of Daily Living (FAAM-ADL) and FAAM-Sports (FAAM-S) subscales, respectively. Participants in the control group had never sustained a LAS. Exclusion criteria for all participants were a history of a lower limb musculoskeletal injury in the 3-month period prior to the study onset, a previous surgery to lower limb musculoskeletal structures, a history of a lower limb fracture that needed realignment and neurological conditions affecting walking biomechanics. If bilateral CAI was reported, the less stable ankle, subjectively decided by participants, was used in the analyses. Prior to their enrolment in the study, all participants provided a written consent to a protocol approved by the Université du Québec à Trois-Rivières Ethics Committee (CER-18-244-07.04).

### Experimental protocol

Before undertaking the clinical gait analysis, participants had to fill the validated French version of the FAAM-ADL, FAAM-S [22] and International Physical Activity Questionnaire (IPAQ) [23] subscales. Participants also had to report the number of previous LAS and the time since the most recent LAS. Participants’ age, height and weight were registered. To quantify participants’ foot morphology, a licensed podiatrist (GM) administered the Foot Posture Index (FPI) [24]. During the clinical gait analysis, lower limb kinematics was recorded using a three-dimensional active motion analysis system (Optotrak Certus, Northern Digital, Waterloo, ON, Canada) at a sampling rate of 100 Hz. Four three-marker clusters were respectively positioned on the sacrum, the distal one third of the thigh, the distal one third of the leg and the posterior part of the calcaneum. The cluster located on the posterior part of the calcaneum was attached using a modified version (to be less brittle and smaller) of the heel plate and wand [25, 26] designed by Telfer et al. [27]. The heel plate was secured on the posterior part of the calcaneum with athletic tape. A standardised hole of 30 mm × 30 mm was cut into the shoes’ heel counter (Rupert model, Athletic Works, China) to allow the insertion of the wand into the heel plate. Fifteen virtual markers, tracked with the marker clusters, were created on the pelvis and tested lower limbs using a digitising pointer on the following anatomical landmarks: bilateral anterior and posterior superior iliac spines, greater trochanter, lateral and medial femoral epicondyles, lateral and medial malleoli, proximal and distal part of the calcaneum posterior surface, sustentaculum tali and fibular tubercle. Ground reaction forces were recorded with a force plate (Bertec Corp, OH, USA) embedded in the floor, at a sampling rate of 2 kHz. Ankle and knee internal moments were calculated using inverse dynamics (synchronised joint kinematics/ground reaction forces and anthropometric data) and resolved in the proximal segment coordinate system.

A wireless surface electromyography (EMG) system (Trigno Wireless; Delsys Inc., Boston, MA, United States) was used to record the activity of gluteus medius, vastus lateralis, gastrocnemius lateralis, fibularis longus and tibialis anterior muscles according to the SENIAM recommendations [28]. The four-bar formation electrodes (27 × 37 × 15 mm) were made of 99% contact material. The interelectrode spacing was 10 mm, the sampling rate was 2 kHz and the gain was 1000. To reduce local impedance over the electrode placement, the skin was shaved, abraded with fine-grade sandpaper and cleaned with alcohol swabs. During the experimentation, a 16 bits A/D converter was used, the common noise removal ratio of the amplifier was > 80 dB and the maximal intraelectrode impedance was 6 kΩ. EMG data were recorded using EMGworks software (Delsys Inc., Boston, MA, United States). Kinematic, kinetic and EMG data were synchronised using Delsys Trigger Module (Delsys Inc., Boston, MA, United States). Walking speed was calculated with electronic photocells timing gates (Brower Timing gate system, USA) positioned 1.35 m before and after the force plate.

During the clinical gait analysis, all participants had to walk at a self-selected speed on a 7.5-m walkway with the force plate located in the centre. First, participants completed five familiarisation trials to calculate their mean walking speed. Then, five walking trials were recorded. The trials during which walking speed exceeded ±5% of the previously determined mean speed or the foot did not entirely strike the force plate were rejected and retaken. During all trials, participants were asked to look straight ahead and not to aim to strike the force plate.

### Data processing

Kinematic, kinetic and EMG data were analysed using Visual 3D software (C-Motion, Germantown, MD, United States). Ankle and knee joint angles and moments were calculated using a X (extension/flexion), Y (adduction/abduction) and Z (internal/external rotation) Cardan sequence. Dual-pass, fourth-order Butterworth low-pass filters with cut-off frequencies of 6 Hz and 50 Hz were used to smooth the marker trajectories and force plate data, respectively. Joint moments were normalised to body mass. EMG data were filtered with a zero lag, bidirectional, 20–450 Hz band-pass fourth-order Butterworth filter. Root Mean Square (RMS) of the raw data were calculated with a 25-ms moving window. To ensure the external validity or our results, EMG data were normalised with the mean quiet stance RMS amplitude for each muscle during the calibration trial. This method was commonly used in previous studies assessing the EMG of the lower limbs during gait in individuals with CAI [17, 29, 30]. Kinematics, kinetics and EMG data were normalised to 100% of the stance phase with 0% representing the initial contact with the force plate and 100% the toe off. Both events were identified with the force plate using a 10 N threshold.

### Statistical analysis

Shapiro-Wilk’s tests were used to evaluate the distribution of the descriptive data (age, weight, height, FPI, mean walking speed, FAAM-S, FAAM-ADL and IPAQ scores). Mann-Whitney U tests were used to compare age, FAAM-ADL, FAAM-S and IPAQ scores between the CAI and control groups as these data were not normally distributed. Independent t tests were used to compare weight, height, FPI scores and mean walking speed between groups. The distribution of EMG, kinematic and kinetic data was evaluated using D’Agostino-Pearson tests. As the data were normally distributed, one-dimensional statistical parametric mapping (independent SPM(t)) was used to compare each individual point of the curves between the CAI and the control groups [31, 32]. Maximum mean between-group differences (MD) and Cohen’s d effect sizes were calculated when statistically significant differences were observed. The threshold of significance was fixed at α = 5% for all analyses. SPM(t) analyses were implemented using the open access SPM1D code (www.spm1d.org) in MATLAB R2020b (The Mathworks Inc., Boston, MA, USA).

## Results

### Demographic and gait speed data

No between-group differences were observed for age (*p* = 0.139), weight (*p* = 0.234), height (*p* = 0.704), FPI (*p* = 0.131), IPAQ scores (*p* = 0.209) and walking speed (*p* = 0.328). FAAM-ADL (*p* < 0.001) and FAAM-S (*p* < 0.001) scores were lower for the CAI compared to the control group (See Table 1).

**Table 1:** Descriptive data

Group	CAI	Control
Gender ratio (M/F)	10/18	9/17
Age (years)	25.5 (±5.5)	23.7 (±4.1)
Weight (kg)	71.3 (±12.3)	67.3 (±12.2)
Height (m)	1.69 (±0.09)	1.70 (±0.09)
Foot posture index	5.1 (±3.1)	3.7 (±3.6)
Last sprain (yr)	1.9 (±1.9)	0.0 (±0.0)
Previous sprains	4.8 (±4.0)	0.0 (±0.0)
FAAM-ADL (%)	83.4 (±8.6)	100.0 (±0.0)^a^
FAAM-Sport (%)	61.9 (±10.6)	99.7 (±1.2)^a^
IPAQ (MET-min/week)	3450 (±3265)	2508 (±2148)
Walking speed (m/s)	1.42 (±0.15)	1.46 (±0.14)

^a^Statistically significant between-group differences

### Kinematic data

**Fig. 1:**
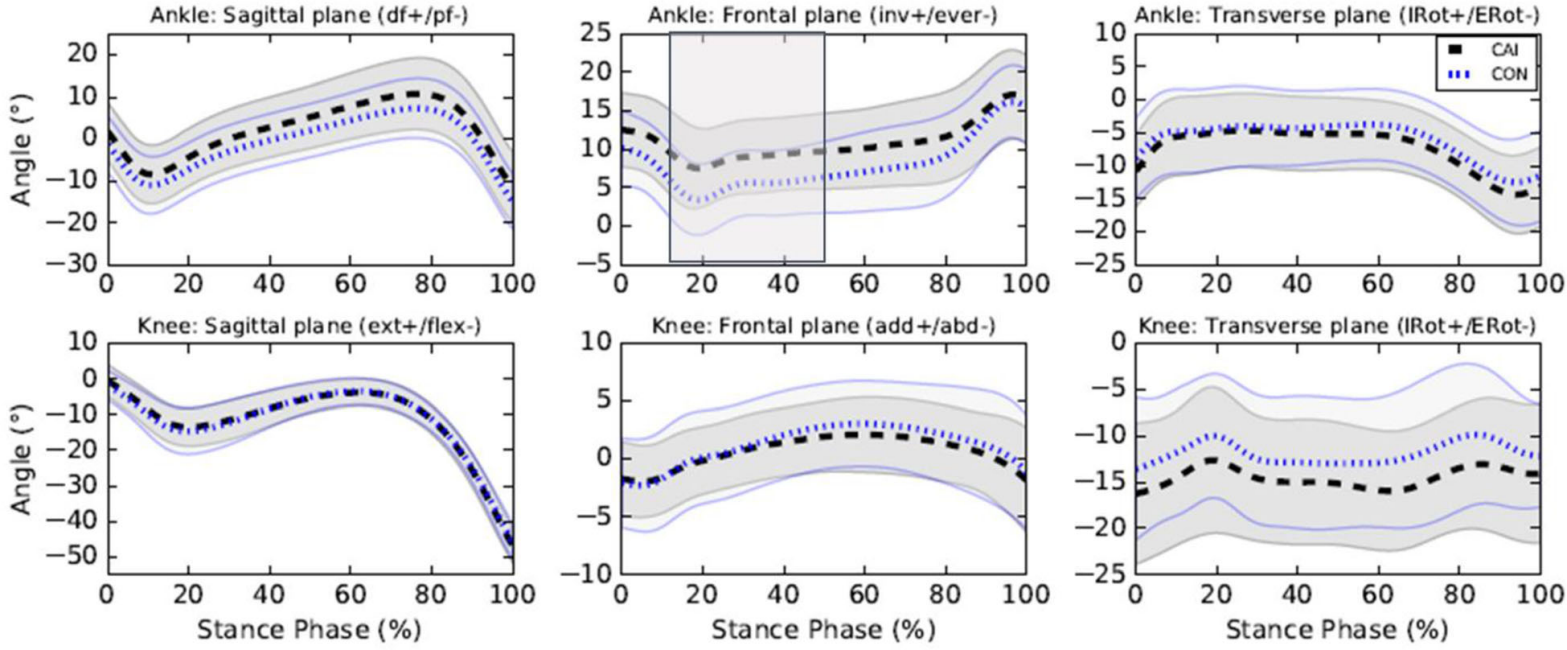
Kinematics of the lower limb during walking. Legends: Means of the CAI (black) and control (CON) (blue) groups are represented by dotted lines and standard deviations are observed between full lines. Significant between-group differences are observed in the shaded region. df: Dorsiflexion, pf: Plantarflexion, inv.: Inversion, ever: Eversion, IRot: Internal rotation, Erot: External rotation, ext.: Extension, flex: Flexion, add: adduction, abd: abduction, IRot: Internal rotation, ERot: External rotation

The CAI group exhibited greater ankle inversion angles from 14 to 48% of the stance phase (%SP) (*p* = 0.008, MD = + 4.10^o^ at 20%SP, d = 0.85 at 22%SP) compared to the control group (see Fig. 1). No between-group differences in ankle sagittal and transverse angles, knee sagittal, frontal and transverse angles were found. The mean differences between the CAI and control groups and the Cohen’s d effect sizes for all biomechanical variables are reported in Additional files 1 and 2, respectively.

### Kinetic data

**Fig. 2:**
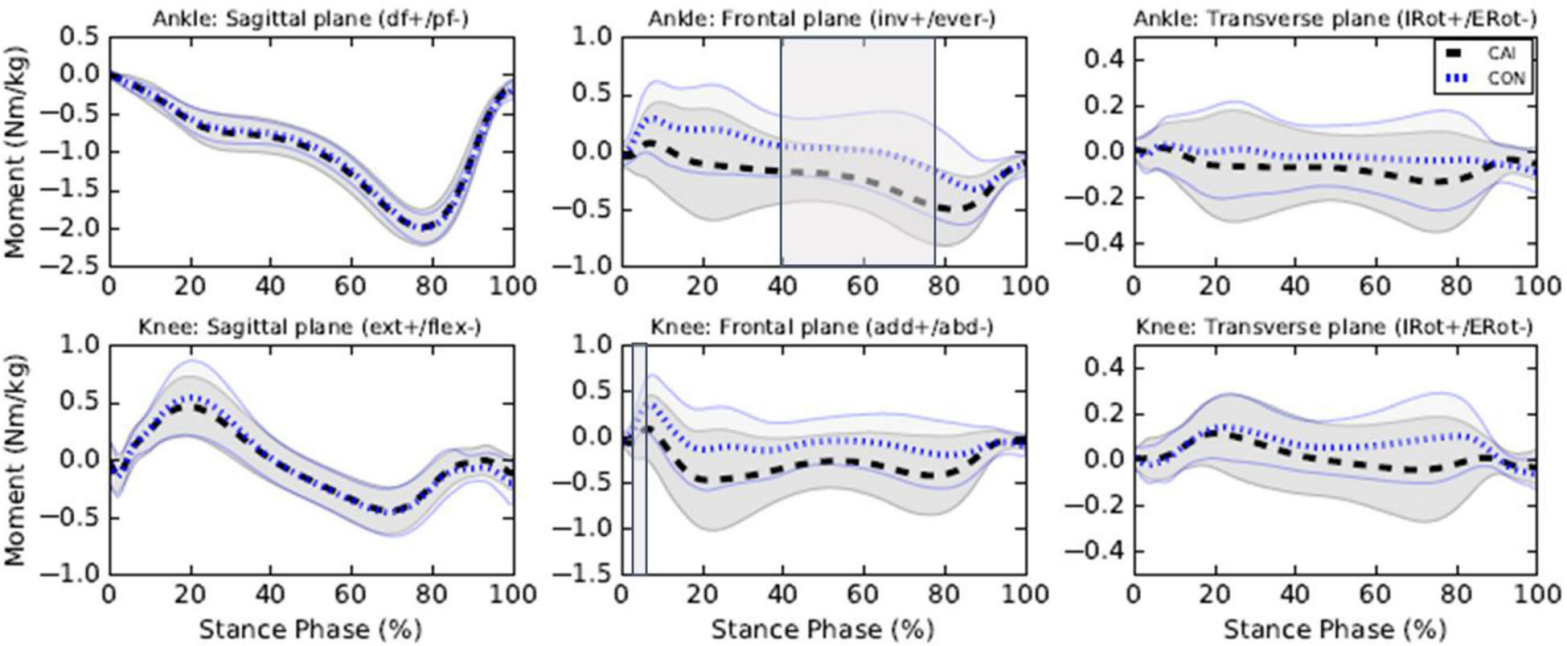
Kinetics of the lower limb during walking. Legends: Means of the CAI (black) and control (CON) (blue) groups are represented by dotted lines and standard deviations are observed between full lines. Significant between-group differences are observed in shaded regions. df: Dorsiflexion, pf: Plantarflexion, inv.: Inversion, ever: Eversion, IRot: Internal rotation, Erot: External rotation, ext.: Extension, flex: Flexion, add: adduction, abd: abduction, IRot: Internal rotation, ERot: External rotation

The CAI group exhibited greater ankle eversion moments from 40 to 78%SP (*p* < 0.001, MD = + 0.32 Nm/kg at 73%SP, d = 0.88 at 63%SP) and greater knee abduction moments from 3 to 6%SP (*p* = 0.045, MD = + 0.23 Nm/kg at 6%SP, d = 0.91 at 5%SP) compared to the control group (see Fig. 2). No between-group differences in ankle sagittal and transverse moments, knee sagittal and transverse moments were found.

### *EMG* data

**Fig. 3:**
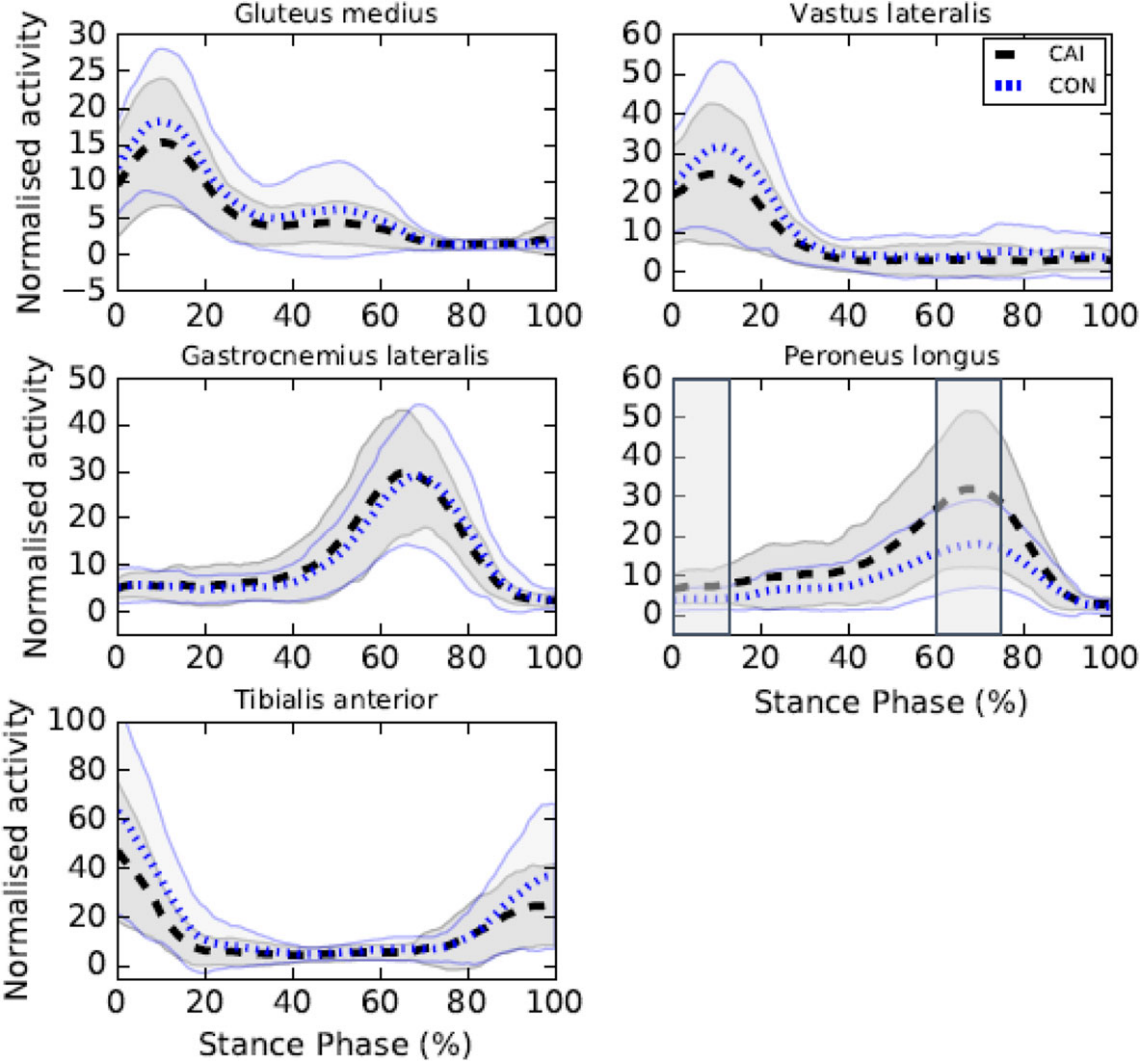
EMG of the lower limb during walking. Legends: Means of the CAI (black) and control (CON) (blue) groups are represented by dotted lines and standard deviations are observed between full lines. Significant between-group differences are observed in shaded regions

The CAI group exhibited greater peroneus longus muscle activity from 0 to 15%SP (*p* = 0.003, MD = + 3.18 at 7%SP, d = 0.89 at 12%SP) and 60 to 76%SP (*p* = 0.003, MD = + 14.10 at 68%SP, d = 0.88 at 66%SP) compared to the control group (see Fig. 3). No between-group differences in gluteus medius, vastus lateralis, gastrocnemius lateralis and tibialis anterior muscle activity were found.

## Discussion

This case-control study aimed to identify lower limb kinematic, kinetic and EMG differences between individuals with CAI and healthy controls during walking. This study was deemed important as only a few studies compared the biomechanics of the lower limbs of individuals with and without CAI using a comprehensive approach including kinematic, kinetic and EMG measures [15, 20, 30]. Using such comprehensive approach allowed the identification of biomechanics adaptations associated with CAI for the same population and investigation of how kinematic, kinetic and EMG adaptations interacted. Also, knee moments differences between individuals with and without CAI during gait were inconsistent and contradictory in previous studies. Indeed, Moisan et al. [20] reported greater and Son et al. [15] reported smaller knee abduction moments in individuals with CAI compared to controls during walking and thus further research was needed to better understand the impact of CAI on this joint. Our main hypotheses were that participants with CAI would exhibit greater ankle inversion angles and peroneus longus muscle activity as well as smaller ankle inversion moments during the stance phase of gait compared to healthy control participants. Our results fully support these hypotheses. The main findings were greater ankle inversion angles, reaching a mean difference of 4.10^o^ at 20%SP (see Additional file 1), in participants with CAI compared to controls which are consistent with previous results [14–16]. However, in contrary to previous findings, no significant differences in ankle inversion angles [15, 33] and moments [15] were observed during the pre-swing phase of gait. Delahunt et al. [14] hypothesised that to prevent excessive ankle inversion movements, the peroneus longus muscle of individuals with CAI activates strongly during the stance phase. Our results are consistent with this hypothesis. Indeed, peroneus longus muscle activity was greater for participants with CAI from 60 to 76%SP, which is about the period during which the between-group differences in ankle frontal movements started to decrease (see Fig. 1). During the pre-swing phase, the ankle frontal movements’ curves of the CAI and control groups nearly overlapped (see Fig. 1), highlighting an efficient biomechanical compensatory strategy in individuals with CAI during the latter half of the stance phase. However, during the first half, participants with CAI could be at greater risk of sustaining a LAS as the ankle is in a more vulnerable position (i.e. more inverted). Indeed, if an external perturbation (e.g., stepping on an uneven terrain) results into increased ankle inversion in individuals with CAI, they would be at more risk of sustaining a LAS as their ankle is already in a more inverted position and their peroneus longus muscle presents a reflex latency and an electromechanical delay of activation [34]. Thus, clinicians should probably focus on the biomechanical deficits during the first part of stance.

Consistent with Monaghan et al. [16] results, participants with CAI exhibited ankle eversion moments whereas controls exhibited ankle inversion moments during most of the stance phase. Even though between-group differences for ankle frontal moments were only statistically significant between 40 to 78%SP, one can observe that both curves are separated from each other for most of the stance phase (5 to 85%SP) (see Fig. 2) with moderate to strong Cohen’s d effect sizes (see Additional file 2). Even if the differences are not statistically different throughout this period, such differences could perhaps be clinically meaningful. Changes in kinetic control of the ankle also represent a protective mechanism to prevent LAS and explain the greater peroneus longus muscle activity [8].

In agreement with previous findings from our research group, participants with CAI exhibited greater knee abduction moments during the early stance [20]. This result suggests that ankle biomechanical changes in the frontal plane induce a knee compensatory mechanism in individuals with CAI. No between-group knee kinematics differences were observed, but the greater knee abduction moments in individuals with CAI could represent an attempt of the locomotor system to attenuate the load and strain on the lateral structures of the ankle using the proximal segment in the kinetic chain. It could also represent an attempt to stabilise the knee during the early stance and to better attenuate the impact forces, known to be greater in individuals with CAI [15]. No between-group difference was observed for the vastus lateralis muscle which is consistent with previous work [20]. However, contrary to our finding, Son et al. [15] reported a decreased vastus lateralis muscle activity of less than 4% in individuals with CAI compared to controls. As the differences were only observed during a period when the activity of the vastus lateralis muscle is rapidly decreasing (i.e., around 18 to 90%SP), the results most likely have little clinical applicability. Further studies investigating the activity of the abductor and adductor muscles are needed to better understand the role of the knee during walking in individuals with CAI. In our study, we observed no between-group differences for the gluteus medius muscle activity, which is consistent with previous results [35, 36]. However, other studies reported greater [17] or lower [15, 20] gluteus medius muscle activity in individuals with CAI compared to controls. The disparity in EMG normalisation methods, dependent variables as well as the different experimental conditions (i.e., shod and barefoot) may perhaps explain the inconsistencies noted in previous results. Further studies are needed to better understand the differences in gluteus medius muscle activity using standardised EMG assessment methods or novel and promising methods such as ultrasound imaging [37].

Our study allows validation and precision of previous results regarding the biomechanics of the lower limb of individuals with CAI during walking, namely the increased ankle inversion angles and moments as well as peroneus longus muscle activity. In clinical contexts, both the cause and consequence of CAI should be addressed. Sensorimotor deficits in motor-neuron pool excitability, reflex reactions, muscular strength and proprioception [38] could be responsible for the changes in gait movement strategy in individuals with CAI [15]. Accordingly, the therapeutic goal in clinical gait rehabilitation should be to address the faulty ankle movements and restore proper sensorimotor function. Impairment-based rehabilitation programs including gait retraining [39, 40] or exercise regimen targeting sensorimotor deficits [41], prescribed independently or together [42], have shown promising results. External feedback during gait retraining reinforces ideal repetitive actions via optimised sensorimotor loops [43] and sensorimotor training allows targeting deficits in both sensory and motor aspects of sensorimotor control [41]. Our results will inform the development of future efficacy trials aiming at determining to what extent addressing the biomechanical particularities of individuals with CAI will result in improved clinical outcomes.

Our results should be interpreted in light of a few limitations. First, forefoot and midfoot segments kinematics were not analysed. Individuals with CAI present kinematic changes for these segments compared to controls during walking [44, 45]. Thus, between-group differences could have been present in our study but could not be observed using our design. Second, as our results suggest that the ankle biomechanical changes induce proximal biomechanical compensations in individuals with CAI, more studies should also investigate between-group differences at the knee and hip joints. This is especially important as it was recently reported that individuals with CAI exhibit a hip-dominant strategy to generate power allowing forward acceleration of the lower limbs during gait [15]. Third, the mean age of the recruited participants was 25.5 and 23.7 years for the CAI and control groups, respectively. Our results could perhaps not be generalisable for older individuals with CAI. Fourth, inherently to the chosen research design, it is unclear if the biomechanical differences found in our study are a cause or consequence of CAI and thus prospective studies are warranted. Fifth, even though the IPAQ scores were not significantly different between groups, high within-group variability was observed. The inclusion of individuals with heterogenous physical activity levels in each group may have contributed to decreasing the homogeneity of our data and thus the ability of our analyses to reach the threshold of significance. Sixth, as we did not include a group of copers, our study does not provide insights regarding the biomechanical differences between individuals who sustained a LAS and healed normally and those who developed CAI. Seventh, even though the biomechanical deficits exhibited by individuals with CAI during overground and treadmill walking are similar [8], our results may perhaps not be entirely generalisable during treadmill walking.

## Conclusions

During the first half of the stance phase of gait, individuals with CAI could be more at risk of sustaining recurrent LAS mostly due to greater ankle inversion angles. However, the greater ankle eversion moments and peroneus longus muscle activity during the second half of the stance phase could be an efficient mechanism to correct this maladaptive gait pattern and allow attenuation of the faulty ankle movements during the pre-swing phase. Rehabilitation protocol should focus on the faulty ankle movements during the initial stance.

## Supplementary Information

**Additional file 1.** Mean differences between the CAI and control groups for all biomechanical variables during the stance phase of gait. Mean differences between the CAI and control groups for all biomechanical variables during the stance phase of gait.

**Additional file 2.** Cohen’s d effect sizes for all biomechanical variables during the stance phase of gait. Cohen’s d effect sizes for all biomechanical variables during the stance phase of gait.

## Data Availability

The datasets used and/or analysed during the current study are available from the corresponding author on a reasonable request.

## Abbreviations

CAI: Chronic ankle instability
CON: Healthy controls
EMG: Electromyography
FAAM-ADL: Foot and Ankle Ability Measures-Activity of Daily Living
FAAM-S: FAAM-Sports
IPAQ: International Physical Activity Questionnaire
LAS: Lateral ankle sprain
RMS: Root Mean Square
SPM: Statistical Parametric Mapping
UQTR: Université du Québec à Trois-Rivières
%SP: Percent of the stance phase

## References

[1] Doherty C, Delahunt E, Caulfield B, Hertel J, Ryan J, Bleakley C (2014). The incidence and prevalence of ankle sprain injury: a systematic review and meta-analysis of prospective epidemiological studies. Sports Med.

[2] Waterman BR, Owens BD, Davey S, Zacchilli MA, Belmont PJ (2010). The epidemiology of ankle sprains in the United States. J Bone Joint Surg Am.

[3] Hubbard-Turner T (2019). Lack of medical treatment from a medical professional after an ankle sprain. J Athl Train.

[4] McKay GD, Goldie PA, Payne WR, Oakes BW (2001). Ankle injuries in basketball: injury rate and risk factors. Br J Sports Med.

[5] Yeung MS, Chan KM, So CH, Yuan WY (1994). An epidemiological survey on ankle sprain. Br J Sports Med.

[6] Doherty C, Bleakley C, Hertel J, Caulfield B, Ryan J, Delahunt E (2016). Recovery from a first-time lateral ankle sprain and the predictors of chronic ankle instability: a prospective cohort analysis. Am J Sports Med.

[7] Hertel J, Corbett RO (2019). An updated model of chronic ankle instability. J Athl Train.

[8] Moisan G, Descarreaux M, Cantin V (2017). Effects of chronic ankle instability on kinetics, kinematics and muscle activity during walking and running: a systematic review. Gait Posture..

[9] Simpson JD, Stewart EM, Macias DM, Chander H, Knight AC. Individuals with chronic ankle instability exhibit dynamic postural stability deficits and altered unilateral landing biomechanics: a systematic review. Phys Ther Sport. 2019;37:210-19. 10.1016/j.ptsp.2018.06.003. 10.1016/j.ptsp.2018.06.003 29914742

[10] Dejong AF, Koldenhoven RM, Hertel J (2020). Proximal adaptations in chronic ankle instability: systematic review and meta-analysis. Med Sci Sports Exerc.

[11] Gribble PA, Bleakley CM, Caulfield BM, Docherty CL, Fourchet F, Fong DT (2016). Evidence review for the 2016 international ankle consortium consensus statement on the prevalence, impact and long-term consequences of lateral ankle sprains. Br J Sports Med.

[12] Hintermann B, Boss A, Schafer D (2002). Arthroscopic findings in patients with chronic ankle instability. Am J Sports Med.

[13] Arnold BL, Wright CJ, Ross SE (2011). Functional ankle instability and health-related quality of life. J Athl Train.

[14] Delahunt E, Monaghan K, Caulfield B (2006). Altered neuromuscular control and ankle joint kinematics during walking in subjects with functional instability of the ankle joint. Am J Sports Med.

[15] Son SJ, Kim H, Seeley MK, Hopkins JT (2019). Altered walking Neuromechanics in patients with chronic ankle instability. J Athl Train.

[16] Monaghan K, Delahunt E, Caulfield B (2006). Ankle function during gait in patients with chronic ankle instability compared to controls. Clin Biomech.

[17] Koldenhoven RM, Feger MA, Fraser JJ, Saliba S, Hertel J (2016). Surface electromyography and plantar pressure during walking in young adults with chronic ankle instability. Knee Surg Sports Traumatol Arthrosc.

[18] Hopkins JT, Coglianese M, Glasgow P, Reese S, Seeley MK (2012). Alterations in evertor/invertor muscle activation and center of pressure trajectory in participants with functional ankle instability. J Electromyogr Kinesiol.

[19] Terada M, Bowker S, Thomas AC, Pietrosimone B, Hiller CE, Rice MS, Gribble PA (2015). Alterations in stride-to-stride variability during walking in individuals with chronic ankle instability. Hum Mov Sci.

[20] Moisan G, Mainville C, Descarreaux M, Cantin V (2020). Kinematic, kinetic and electromyographic differences between young adults with and without chronic ankle instability during walking. J Electromyogr Kinesiol.

[21] Gribble PA, Delahunt E, Bleakley CM, Caulfield B, Docherty CL, Fong DT (2014). Selection criteria for patients with chronic ankle instability in controlled research: a position statement of the international ankle consortium. J Athl Train.

[22] Borloz S, Crevoisier X, Deriaz O, Ballabeni P, Martin RL, Luthi F (2011). Evidence for validity and reliability of a French version of the FAAM. BMC Musculoskelet Disord.

[23] Criniere L, Lhommet C, Caille A, Giraudeau B, Lecomte P, Couet C (2011). Reproducibility and validity of the French version of the long international physical activity questionnaire in patients with type 2 diabetes. J Phys Act Health.

[24] Redmond AC, Crosbie J, Ouvrier RA (2006). Development and validation of a novel rating system for scoring standing foot posture: the foot posture index. Clin Biomech.

[25] Moisan G, Mainville C, Descarreaux M, Cantin V (2020). Unilateral jump landing neuromechanics of individuals with chronic ankle instability. J Sci Med Sport.

[26] Moisan G, Mainville C, Descarreaux M, Cantin V (2019). Effects of foot orthoses on walking and jump landing biomechanics of individuals with chronic ankle instability. Phys Ther Sport..

[27] Telfer S, Abbott M, Steultjens MPM, Woodburn J (2013). Dose-response effects of customised foot orthoses on lower limb kinematics and kinetics in pronated foot type. J Biomech.

[28] Hermens HJ, Freriks B, Disselhorst-Klug C, Rau G (2000). Development of recommendations for SEMG sensors and sensor placement procedures. J Electromyogr Kinesiol.

[29] Koldenhoven RM, Feger MA, Fraser JJ, Hertel J (2018). Variability in center of pressure position and muscle activation during walking with chronic ankle instability. J Electromyogr Kinesiol.

[30] Koldenhoven RM, Hart J, Saliba S, Abel MF, Hertel J (2019). Gait kinematics & kinetics at three walking speeds in individuals with chronic ankle instability and ankle sprain copers. Gait posture.

[31] Pataky TC, Vanrenterghem J, Robinson MA (2015). Zero- vs. one-dimensional, parametric vs. non-parametric, and confidence interval vs. hypothesis testing procedures in one-dimensional biomechanical trajectory analysis. J Biomech.

[32] Pataky TC, Vanrenterghem J, Robinson MA (2016). The probability of false positives in zero-dimensional analyses of one-dimensional kinematic, force and EMG trajectories. J Biomech.

[33] Drewes LK, McKeon PO, Paolini G, Riley P, Kerrigan DC, Ingersoll CD (2009). Altered ankle kinematics and shank-rear-foot coupling in those with chronic ankle instability. J Sport Rehabil.

[34] Hoch MC, McKeon PO (2014). Peroneal reaction time after ankle sprain: a systematic review and meta-analysis. Med Sci Sports Exerc.

[35] Kautzky K, Feger MA, Hart JM, Hertel J (2015). Surface electromyography variability measures during walking: effects of chronic ankle instability and prophylactic bracing. Athletic training &amp. Sports Health Care.

[36] Feger MA, Donovan L, Hart JM, Hertel J (2015). Lower extremity muscle activation in patients with or without chronic ankle instability during walking. J Athl Train.

[37] DeJong AF, Mangum LC, Hertel J (2019). Gluteus medius activity during gait is altered in individuals with chronic ankle instability: an ultrasound imaging study. Gait & Posture.

[38] Hertel J (2008). Sensorimotor deficits with ankle sprains and chronic ankle instability. Clin Sports Med.

[39] Torp DM, Thomas AC, Donovan L (2019). External feedback during walking improves measures of plantar pressure in individuals with chronic ankle instability. Gait Posture.

[40] Donovan L, Feger MA, Hart JM, Saliba S, Park J, Hertel J (2016). Effects of an auditory biofeedback device on plantar pressure in patients with chronic ankle instability. Gait Posture..

[41] McKeon PO, Wikstrom EA (2016). Sensory-targeted ankle rehabilitation strategies for chronic ankle instability. Med Sci Sports Exerc.

[42] Donovan L, Hart JM, Saliba S, Park J, Feger MA, Herb CC, Hertel J (2016). Effects of ankle destabilization devices and rehabilitation on gait biomechanics in chronic ankle instability patients: a randomized controlled trial. Phys Ther Sport..

[43] Huang H, Wolf SL, He J (2006). Recent developments in biofeedback for neuromotor rehabilitation. J Neuroeng Rehabil.

[44] De Ridder R, Willems T, Vanrenterghem J, Robinson M, Pataky T, Roosen P (2013). Gait kinematics of subjects with ankle instability using a multisegmented foot model. Med Sci Sports Exerc.

[45] Wright CJ, Arnold BL, Ross SE, Pidcoe PE (2013). Individuals With Functional Ankle Instability, but not Copers, Have Increased Forefoot Inversion During Walking Gait. Athl Train Sports Health Care.

