# Supplementary figures and images for: Lower limb biomechanics in individuals with chronic ankle instability during gait: a case-control study
Source: J Foot Ankle Res. 2021 May 3;14:36. doi: 10.1186/s13047-021-00476-6 (PMC8091674; doi:10.1186/s13047-021-00476-6)

Mean differences (+: CAI>CON)

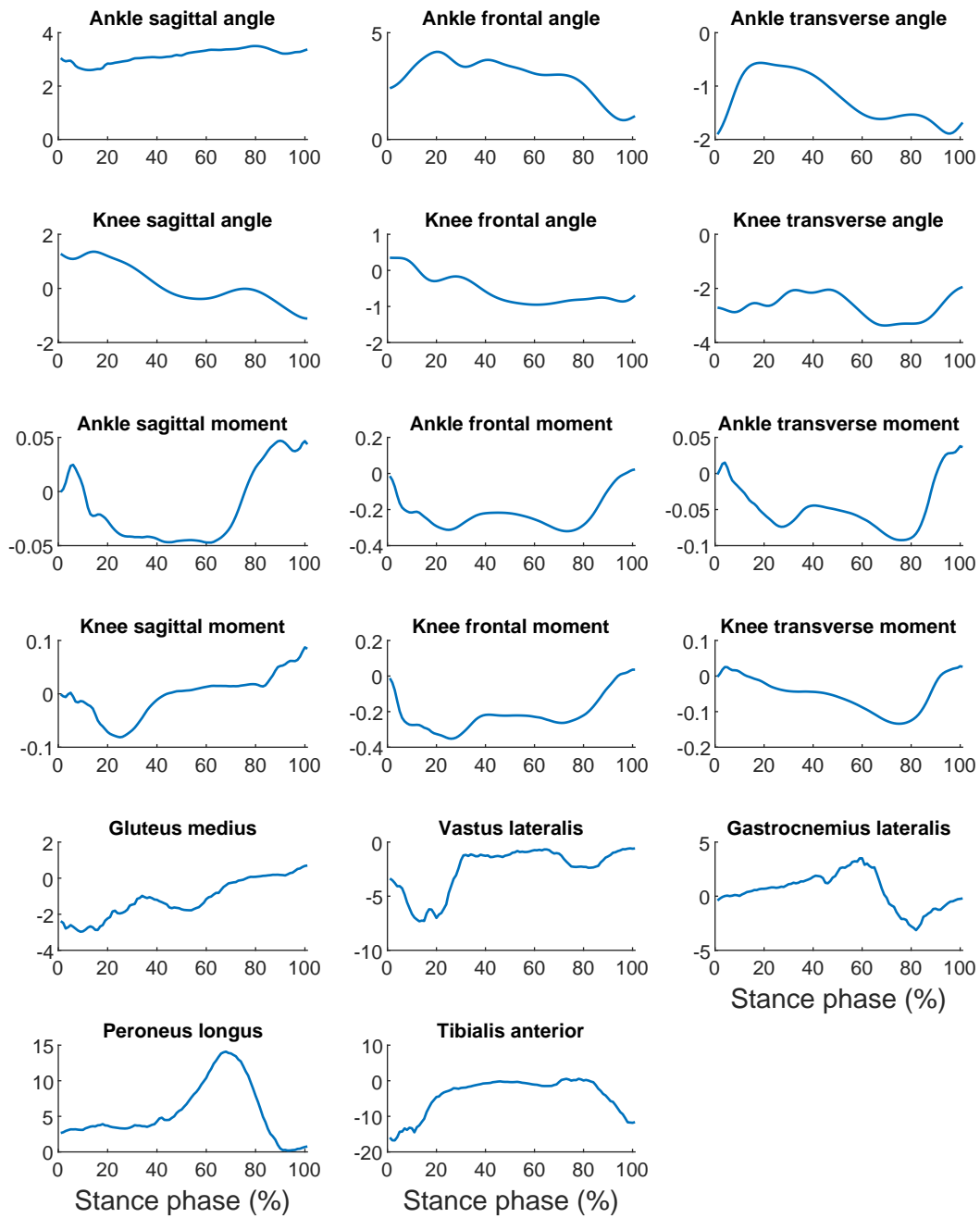

Supplement: Supplementary file 1 — Additional file 1. Mean differences between the CAI and control groups for all biomechanical variables during the stance phase of gait. Mean differences between the CAI and control groups for all biomechanical variables during the stance phase of gait. [file 13047_2021_476_MOESM1_ESM.pdf]

Cohen's d effect size

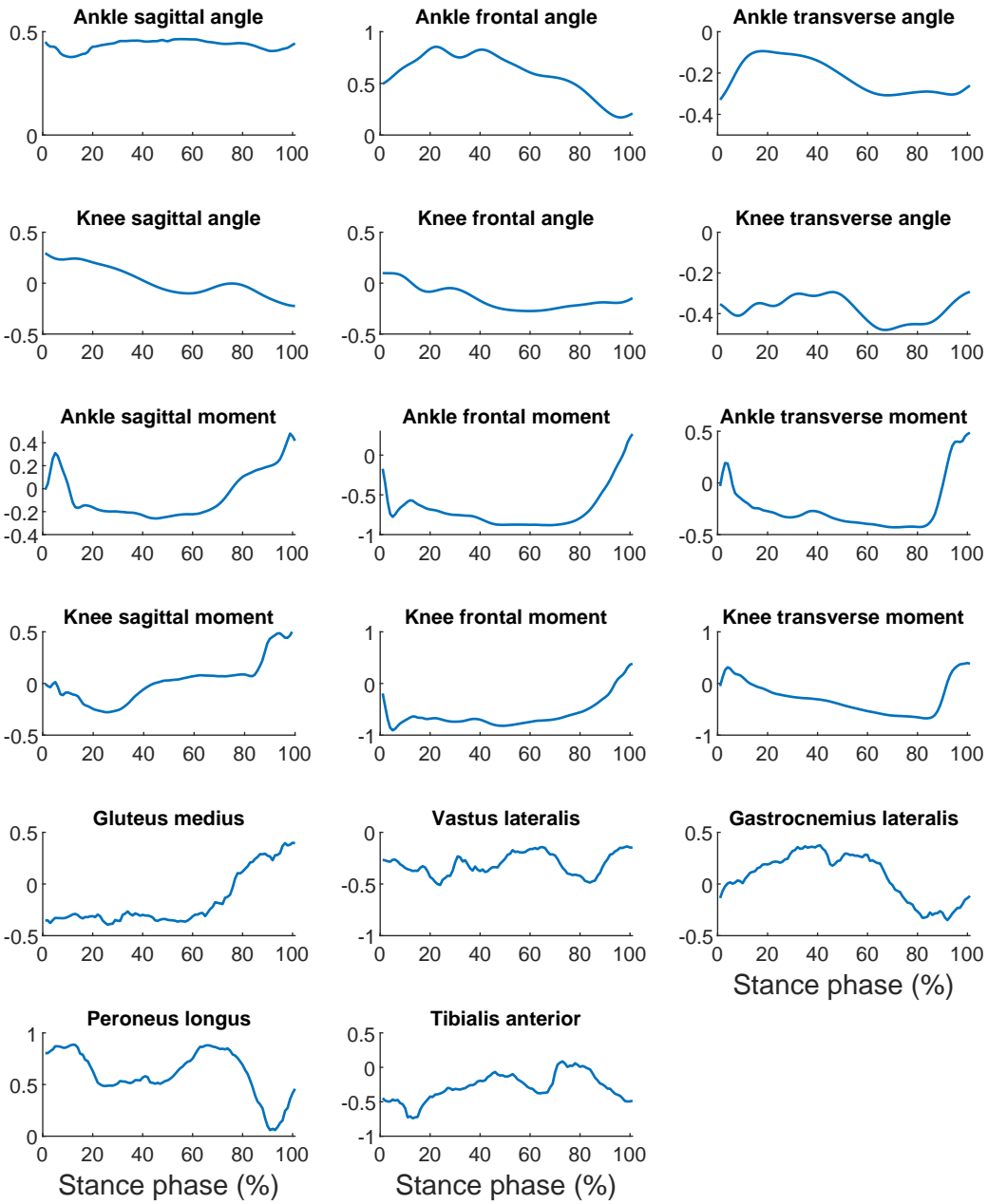

Supplement: Supplementary file 2 — Additional file 2. Cohen’s d effect sizes for all biomechanical variables during the stance phase of gait. Cohen’s d effect sizes for all biomechanical variables during the stance phase of gait. [file 13047_2021_476_MOESM2_ESM.pdf]
